# Current use and development of monoclonal antibodies for the treatment of systemic lupus erythematosus: a review

**DOI:** 10.1093/abt/tbae033

**Published:** 2024-12-26

**Authors:** Alexander Blagov, Nikolay Orekhov, Alexey Churov, Irina Starodubtseva, Dmitry Beloyartsev, Tatiana Kovyanova, Vasily Sukhorukov, Alexander Orekhov

**Affiliations:** Laboratory of Angiopathology, Institute of General Pathology and Pathophysiology, 8 Baltiiskaya Street, Moscow 125315, Russia; Laboratory of Angiopathology, Institute of General Pathology and Pathophysiology, 8 Baltiiskaya Street, Moscow 125315, Russia; Laboratory of Angiopathology, Institute of General Pathology and Pathophysiology, 8 Baltiiskaya Street, Moscow 125315, Russia; Institute on Aging Research, Russian Gerontology Clinical Research Centre, Pirogov Russian National Research Medical University, 16, 1st Leonova Street, Moscow 129226, Russia; Department of Polyclinic Therapy, NN Burdenko Voronezh State Medical University, 10 Studencheskaya Street, Voronezh 394036, Russia; Vascular Surgery Department, A. V. Vishnevsky National Medical Research Center of Surgery, 27 Bolshaya Serpukhovskaya Street, Moscow 117997, Russia; Laboratory of Angiopathology, Institute of General Pathology and Pathophysiology, 8 Baltiiskaya Street, Moscow 125315, Russia; Institute for Atherosclerosis Research, Osennyaya Street 4-1-207, Moscow 121609, Russia; Laboratory of Angiopathology, Institute of General Pathology and Pathophysiology, 8 Baltiiskaya Street, Moscow 125315, Russia; Laboratory of Angiopathology, Institute of General Pathology and Pathophysiology, 8 Baltiiskaya Street, Moscow 125315, Russia

## Abstract

The development of targeted drugs for the treatment of systemic lupus erythematosus (SLE) is a promising area of research because targeted drugs are associated with a lower risk of severe side effects than systemic drugs. There are only two approved drugs based on monoclonal antibodies (a group of targeted drugs) for the treatment of SLE, so there is an unmet need for the development of new and improved antibody analogs. This review analyzes the effectiveness and safety of both already approved antibodies (anifrolumab and belimumab) for the treatment of SLE and antibodies under development with an assessment of their future prospects for entering the pharmaceutical market. In addition to the antibodies themselves, the choice of their therapeutic targets and what role the targets can play in the effectiveness and safety of the antibodies are analyzed here.

## Teaser

**Statement of Significance**

- Among FDA-approved mAbs, anifrolumab is more effective than belimumab, but the latter is not associated with the development of opportunistic infections.
- There are several potential antibody developments for the treatment of SLE, whose entry into the clinic is possible within 5–10 years: obinutuzumab to CD20, AMG557 to ICOS, dopyrolizumab pegol to CD40L, and tolizumab to IL6R.

## Introduction

Systemic lupus erythematosus (SLE) is a chronic autoimmune disease that affects multiple organ systems with a characteristic pattern of exacerbations and remissions [1]. In SLE, damage to the joints, skin, central nervous system, kidneys, and blood vessels can occur [2]. The etiology of SLE still remains not fully understood; the most likely route of occurrence of SLE is the close relationship of a number of environmental factors with genetic determinants, which leads to the launch of an autoimmune reaction, manifested in increased production of pathogenic autoantibodies by B cells and a “burst” of cytokine reactions, which ultimately leads to damage to tissues and organs [1].

SLE is more than eight times more common in women than men, with a global prevalence of 3.04 million in women compared to 0.36 million in men [3]. In this case, the main risk group is women of childbearing age, for whom the development of SLE is often associated with the emergence of various psychosocial problems that can affect family planning and pregnancy [1].

A complete cure for SLE is not possible today, but the treatment options used help improve the condition of patients and reduce the aggravation of further development of SLE. At the initial stage of the disease, in the absence of severe organ damage, the anti-malarial drug hydroxychloroquine is used to treat SLE [4]. Low cost and long-term safety make it one of the cornerstone drugs for the treatment of SLE, but, in cases of severe disease, hydroxychloroquine is not very effective [5]. For the treatment of moderate and severe SLE, glucocorticoids are used to promote rapid remission, but their long-term use is limited due to high toxicity [4]. Taking steroids in some situations can be replaced by immunosuppressants, such as azathioprine, methotrexate, mycophenolate mofetil, and cyclophosphamide, which, however, also have significant side effects [5]. Monoclonal antibodies, despite their drawbacks, including the development of serious allergic reactions and reactogenicity associated with the formation of antidrug antibodies (ADA) [6], are an additional effective means of combating SLE. Due to the fact that monoclonal antibodies are targeted therapy, they have limited adverse reactions in comparison with the above-mentioned drugs for systemic therapy.

In this review, the following aims had been suggested: the first aim is to identify promising antigenic targets for the development of future monoclonal antibodies for the treatment of SLE and analyze the existing target proteins (why they were chosen). Second, it is to analyze the effectiveness and safety of already approved antibodies for the treatment of SLE and monoclonal antibodies under development (for what reasons they may or may not complement existing therapy). Third, it is to propose ways in which the efficacy and safety of both new and approved antibodies for the treatment of SLE can potentially be improved.

## Used antigenic targets for the treatment of systemic lupus erythematosus

Currently, the Food and Drug Administration (FDA) has approved two monoclonal antibodies for the treatment of SLE: anifrolumab, which targets the interferon I receptor, and belimumab, which targets B-cell activating factor (BAFF). An important factor in the therapeutic effectiveness of antibodies is the choice of antigen and even specific epitopes in its structure to which the monoclonal antibody binds. This section will analyze the key features of these antigens (interferon I receptor and BAFF), the inactivation of which helps to alleviate the course of the SLE disease.

### Interferon I receptor

Interferon I receptor subunit 1 was chosen as a therapeutic target for anifrolumab and not by chance [5]. It is known that increased levels of type I interferons are observed in SLE and are directly related to disease activity [7]. Based on a number of studies, it is safe to say that type I interferons, and especially interferon-alpha (INFα), play a central role in the pathogenesis of SLE [8–10]. Its central role is due to the regulation of multiple immune pathways affecting the maturation and proliferation of leukocytes, as well as the development of an aberrant immune response. Thus, interferon I in SLE, through its effect on myeloid dendritic cells, activates the priming and differentiation of autoimmune T cells, also through mDC, and it directly stimulates the maturation of autoimmune B cells through the regulatory proteins BAFF and APRIL, activates NETosis by neutrophils, and enhances phagocytic and cytotoxic effects in macrophages and natural killer (NK) cells [11]. Thus, blocking the binding of interferon I to its receptor may “turn off” the main pathological signaling pathways in SLE. The main producers of interferon I are plasmacytoid DCs (pDCs), which are activated to transcribe the interferon I gene after interaction with immune complexes consisting of autoantibodies and nucleic acid from destroyed cells [12]. Other immune cells, such as macrophages and NK and B lymphocytes, also take part in the activation of interferon production by pDCs, which creates a positive feedback for the development of an autoimmune reaction in SLE [11]. In addition, interferon I has been suggested to influence the development of renal complications in patients with SLE [11].

### B-cell activating factor

BAFF is the therapeutic target of the approved monoclonal antibody belimumab, as well as a number of other antibodies in development [5]. BAFF is one of the key regulators of B-lymphocyte maturation. BAFF, located on the surface of mDC, in response to the production of type I interferon, binds to three receptors that are expressed by B cells: BAFF-R, TACI, and BCMA, which leads to the maturation of autoreactive B cells [13]. A study [14] showed that BAFF expression is particularly upregulated in atypical B-cell subsets responsible for the development of SLE. As can be seen, in contrast to interferon I, targeting BAFF represents a narrower intervention in the pathological process, affecting predominantly the humoral autoimmune response, which, on the one hand, can help reduce possible side effects for a potential antibody but, on the other hand, may not be as effective. An additional difficulty when choosing BAFF as a biological target is the fact that this protein is multimerized [13], which may require a larger amount of antibodies to completely neutralize BAFF signaling.

## Analysis of antibodies approved for the treatment of systemic lupus erythematosus

### Anifrolumab

Anifrolumab is one of two currently approved antibodies against SLE. Anifrolumab is a pure human immunoglobulin G1 aimed at inhibiting the activation of the interferon cascade by binding to subunit 1 of the type I IFN receptor [5]. In particular, due to these properties, anifrolumab has successfully passed clinical trials. As noted above: The type-I interferon (IFNI) is a central signaling link in the pathogenesis of SLE, and its neutralization is expected to help block various autoimmune cascades in SLE. The fact that anifrolumab is a fully human antibody helps reduce the likelihood of serious side effects.

FDA approval was based on efficacy and safety data from the Phase IIb MUSE study (300 and 1000 mg and placebo arms) and the TULIP-1 (150 or 300 mg or placebo) and Phase III 2 studies (300 mg or placebo) in adult patients with moderate to severe SLE [5]. All patients, both in the control and placebo groups, also received standard therapy, including glucocorticoids and immunosuppressants, while the dose of glucocorticoids (prednisolone) was reduced during clinical trials [15]. The phase IIb MUSE study met the primary endpoint of the percentage of patients achieving systemic lupus erythematosus response index-4 (SRI-4) in the 300 mg (34.3%) and 1000 mg (28.8%) groups significantly responded to treatment compared to placebo (17.6%) [15]. The TULIP-1 study did not meet the SRI-4-based primary endpoint, but there were a number of benefits: several patients achieved secondary endpoint targets, as well as a high level of suppression of the 21-gene type I IFN pharmacodynamic signature (>80 %) [15]. In the TULIP-2 phase III clinical trial, the primary endpoint was met based on the British Isles Lupus Assessment Group-based Composite Lupus Assessment (BICLA) response at week 52: 47.8% in the anifrolumab group versus 31.5% in the placebo group, as well as all secondary endpoints. Also in TULIP-2, anifrolumab demonstrated significant improvements in cutaneous manifestations of SLE based on the CLASI score [15]. The incidence of adverse events (AEs) was 85% to 89% in the anifrolumab groups versus 77% to 84% in the placebo groups. The most common AEs were upper respiratory tract infections (from 12% to 20%) [15]. An increased incidence of herpes zoster was observed in the anifrolumab groups (5%–7%) compared with placebo groups (1%–2%), although most cases were mild and did not lead to treatment discontinuation [15].

An important benefit of taking anifrolumab is the elimination of the need for glucocorticoids in patients with SLE, which helps reduce the overall number of side effects from combination therapy. Thus, ~90% of patients who took anifrolumab showed a decrease in disease activity without the need to start or continue glucocorticoid therapy [16]. Based on a retrospective clinical study, anifrolumab was shown to be a more clinically effective drug compared to another approved antibody for the treatment of SLE, belimumab: based on the DAI (Disease Activity Index), anifrolumab showed a 2-fold greater likelihood of reducing disease activity compared to belimumab, making it the most effective antibody-based targeted biologic for the treatment of SLE [17].

### Belimumab

Belimumab, a monoclonal antibody against BAFF, was approved for the treatment of SLE based on the results of the phase III clinical trials BLISS-52 and BLISS-76 [5]. Both studies were conducted in adult volunteers—patients with antinuclear body-positive SLE who were simultaneously receiving standard therapy. Patients were divided into groups: placebo, belimumab at a dose of 10 mg/kg, and belimumab at a dose of 1 mg/kg [5]. At week 52, the BLISS-52 study endpoints were met: SRI-4 response was more likely to be achieved in patients receiving belimumab compared with placebo (57.6%—10 mg/kg belimumab and 51.4%—1 mg/kg belimumab versus 43.6% in the placebo group) [18]. Similarly, the efficacy of belimumab was shown in the BLISS-76 study: (43.2% and 40.6% versus 33.5%, respectively) for the primary endpoint and additionally for the secondary endpoint at week 76 of the study: (38.5% versus 39.1% versus 32.4%, respectively). At the same time, a reduction in the dose of corticosteroids by week 72 of the study occurred in 27.7% of patients taking belimumab at a dose of 1 mg/kg, and in 25.8% of patients taking belimumab at a dose of 10 mg/kg [19]. The overall incidence of AEs in both studies, including the placebo group, was 92%, with the most common AEs being headache (16 to 23%) and upper respiratory tract infections (12 to 19%) [18], [19]. Rare, severe AEs occurred with equal frequency in all groups and were not associated with belimumab [18], [19]. Also in the PLUTO trial, belimumab was approved for the treatment of children aged 5 to 17 years [20].

Overall, belimumab showed a similar safety profile compared with anifrolumab, with the exception of no increased incidence of herpes zoster, suggesting a possible lower risk of developing mild opportunistic infections with belimumab. However, as already noted, belimumab was less effective than anifrolumab in a retrospective study. The efficacy and safety of both drugs were independent of the dose administered, suggesting that using the lowest dose tested was appropriate. The approval of belimumab for children makes it the only antibody available to date that can be used to treat pediatric SLE. A comparison of both antibodies is shown in Table 1.

**Table 1:** Antibodies approved for the treatment of SLE: comparison and analysis

**Antibody**	**Anifrolumab**	**Belimumab**
Target	IFNI receptor	BAFF
Efficiency	1. The DAI-based belimumab is two times more effective, which can, in particular, be explained by inhibition of the IFNI signaling cascade, which leads to an initial blocking of many pathological pathways, including both cellular and humoral autoimmune responses. 2. Taking anifrolumab can completely eliminate the need for corticosteroids.	1. The lower efficacy compared to anifrolumab is due, in part, to its targeting of BAFF, which blocks the maturation of autoimmune B cells without affecting other autoimmune processes. 2. Taking belimumab can reduce the use of corticosteroids.
Safety	Mild to moderate AEs. The most common AEs are associated with upper respiratory tract infections (up to 20%), as well as an increased incidence of opportunistic infections, which may be due to greater suppression of the immune system due to blocking IFNI signaling.	Mild to moderate AEs. The most common AEs are associated with upper respiratory tract infections (up to 20%); no opportunistic infections were observed. It showed a slightly better safety profile compared to anifrolumab, which may be due to less immunosuppression.
Future prospects	It is advisable to conduct clinical trials in pediatric patients since the presence of the only approved monoclonal antibody for the treatment of SLE (belimumab) makes this group of patients still highly vulnerable.	It is promising to think about modifications to the belimumab molecule that will reduce the immunogenicity and increase the safety of this antibody. This will make it more likely to prescribe belimumab to patients who have contraindications for taking this group of drugs.

## Potential antigenic targets for the treatment of systemic lupus erythematosus

The development of new effective monoclonal antibodies could significantly complement already approved therapies for the treatment of SLE. For this reason, there is a search for new antigenic targets that are directly involved in the pathogenesis of SLE.

### B-cell surface antigens

Targeting B-cell signaling is one of the popular avenues for developing antibodies for the treatment of SLE. In addition to extracellular B-cell activators, such as the previously discussed BAFF, B-cell surface antigens such as CD20, CD22, and CD19 have also been proposed as therapeutic targets [21].

The CD20 and CD22 receptors are expressed on the surface of immature and mature B lymphocytes and are not expressed on the surface of plasma cells [21]. On the one hand, targeting these antigens makes it possible to reduce the risk of developing severe side effects, primarily associated with the development of infectious diseases. However, on the other hand, the high specificity of these antigens may reduce the effectiveness of therapy. In particular, the disapproval of rituximab (a monoclonal antibody targeting CD20) for the treatment of SLE is thought to be related to the proliferation of long-lived autoimmune plasma cells [21]. The function of CD20 is thought to be associated with the activation of B-cell activity, and CD22 is clearly an inhibitory co-receptor for the BCR, preventing overstimulation of B cells [21]. Thus, antibodies directed against CD22 should have an agonistic effect. It is known that in patients with SLE there are a number of mutations that limit the functions of CD22 [22], which may explain the insufficient effectiveness of the antibodies being developed, in the case of a high proportion of such mutations in the group of participants in clinical trials. CD19, unlike CD20 and CD22, is expressed not only on B cells but also on plasma cells, which not only potentially contributes to better effectiveness of antibodies directed at it but also implies greater safety risks [21].

### T-cell surface antigens

Due to the involvement of T lymphocytes in the activation of both cellular and humoral autoimmune responses, inhibition of their action may have more effective results compared with the strategy of targeting B-lymphocyte antigens. Surface therapeutic targets focused on T lymphocytes include, in particular, proteins such as CD44, Inducible T Cell Costimulator (ICOS), and CD40L.

CD44 is expressed in different cell types, and in SLE its increased expression is noted in both T lymphocytes and B lymphocytes [23], [24]. CD44 is involved in T-cell adhesion and migration [23] and can also potentially participate in the activation and proliferation of B cells through the HA-CD44-AIM2 signaling pathway [24]. As a result of alternative splicing, a large number of isoforms of the CD44 protein are formed, while the CD44v3 and CD44v6 isoforms have a pathological role in SLE [25]. Researchers should keep this in mind when conducting assays to assess the affinity of antibodies under development.

ICOS is a surface antigen that is overexpressed on T follicular helper cells (TFH) [23]. It has been shown that the ratio of effector TFH to regulatory TFH increases in patients with SLE, which may be associated with impaired T-cell differentiation [26]. ICOS is involved in the generation of the ICOS-B7RP1 signaling pathway, which ensures the differentiation of B lymphocytes and activates T-lymphocyte-dependent production of autoantibodies [23].

CD40L is overexpressed on activated CD4+ T lymphocytes [23]. Of primary interest is the interaction of CD40L with its receptor CD40 on B lymphocytes and other immune cells in the renal interstitium, which leads to the development of severe inflammation in both the interstitium and the glomeruli of the kidneys and the subsequent development of lupus nephritis [27]. Thus, targeting the CD40L–CD40 pathway has the potential to prevent renal complications in SLE patients.

### Cytokines

With the successful completion of phase 3 clinical trials of anifrolumab, which targets the IFN I receptor, it is prudent to investigate the effect of blocking other inflammatory pathways associated with cytokine action. Cytokines such as IL17, IL23, and IL6 can be considered as potential therapeutic targets in this case.

IL17 is one of the key markers of human autoimmune diseases, including SLE [28]. The main producers of IL17 are the population of Th17 T lymphocytes, which, in addition to IL17, produce a number of other cytokines, which contributes to increased autoimmune inflammation [28]. IL17 is capable of influencing both the humoral and cellular branches of the autoimmune response: on the one hand, it stimulates the activation of B lymphocytes, which leads to the production of autoantibodies, and, on the other hand, it promotes the recruitment of monocytes and neutrophils to the site of inflammation and infiltration of T cells. In both cases, this ultimately leads to increased inflammation and organ damage [29].

The level of IL23, as well as the level of IL17, correlates with the severity of the development of autoimmune diseases, including SLE [30]. IL23 is a major regulator of Th17 T-cell maturation, and it also directly influences IL17 production [31]. Thus, blocking IL17 will potentially inhibit all signaling pathways associated with IL17 activity. In this case, two subunits of this heteromeric protein can be considered as targets: IL-12p40 and IL-23p19 [32].

IL6 has multiple systemic inflammatory effects. It is able to stimulate plasma cells to increase the production of autoantibodies, as well as stimulate the secretion of IL17, suppress the maturation of Treg lymphocytes, and promote increased local inflammation in affected tissues [33], [34]. In addition, IL6 is responsible for the development of lupus nephritis [35]. Its broad impact in SLE makes IL6 a potentially promising therapeutic target.

## Analysis of antibodies being developed for the treatment of systemic lupus erythematosus

### Antibodies targeting B-cell surface antigens

Rituximab, a monoclonal antibody that targets the CD20 antigen located on the surface of T cells, is approved for the treatment of another autoimmune disease, rheumatoid arthritis. It would be promising to use retuximab for the treatment of other autoimmune diseases, including SLE, but, according to the results of the EXPLORER [36] and LUNAR [37] clinical trials assessing the effectiveness and safety of rituximab for the treatment of moderate and severe SLE (in combination with prednisone), lupus nephritis (in combination with mycophenolate mofetil and corticosteroids) did not meet the primary and secondary endpoints. Despite this result, rituximab showed a number of positive therapeutic effects, such as: clinical efficacy shown in the subgroup of African Americans and Hispanics in the EXPLORER study [36]; according to the results of the LUNAR study, proteinuria was improved, as well as serum complement C3, C4, and antidouble-stranded DNA levels [37], which gives some hope for continuing the search for the use of rituximab in SLE. The incidence of AEs in the study and placebo groups was similar in both studies, with the most common AEs related to infectious diseases [36, 37]. Due to the chimeric nature of rituximab, its administration may be complicated by the development of a humoral response caused by the appearance of human antichimeric antibodies [21].

The ineffectiveness of rituximab in the treatment of SLE is associated with the inability to completely destroy autoimmune B lymphocytes, as a result of which remaining B cells in the affected tissues contribute to further increased inflammation [38]. In this regard, other monoclonal antibodies to CD20 were developed: ocrelizumab and obinutuzumab, which showed significantly better efficacy in depleting autoimmune B lymphocytes compared to rituximab [39], [40]. Their additional advantage is the fact that they are completely humanized, which reduces the risk of developing allergic reactions [21]. However, despite the reported efficacy of ocrelizumab, the phase III study was discontinued due to the development of severe side effects associated with ocrelizumab. These side effects were associated with the development of opportunistic infections and included: pneumonia, herpes zoster, cryptococcal meningitis, and systemic herpes, and eight deaths were reported in the ocrelizumab group [41]. Obinutuzumab showed more encouraging results in the phase II NOBILITY study of lupus nephritis: 35% of obinutuzumab-treated patients met the primary endpoint, and obinutuzumab was not associated with an increased incidence of serious adverse events [42]. In view of this, obinutuzumab is one of the leading candidates as a potential targeted therapy for SLE targeting CD20.

Epratuzumab is a monoclonal antibody designed to enhance CD22 signaling, a negative regulator of B-cell activation. In phase III studies (EMBODY-1 and EMBODY-2), epratuzumab failed to meet the primary endpoints despite symptomatic disease improvement [43]. No serious side effects associated with epratuzumab were reported [43]. Clinical studies of the efficacy and safety of obexelimab, which targets CD19, showed similar results [44]. The absence of serious side effects gives hope for resuming studies of these antibodies, possibly with other target groups of subjects. A comparison of antibodies in development targeting B-cell surface antigens is shown in Table 2.

**Table 2:** Antibodies in development for the treatment of SLE targeting B-cell surface antigens: comparison and analysis

**Antibody**	**Rituximab**	**Ocrelizumab**	**Obinutuzumab**	**Epratuzumab**	**Obexelimab**
Target	CD20			CD22	CD19
Efficiency	Primary and secondary endpoints were not achieved. Improvement in a number of clinical parameters has been shown for some groups of patients. The reason for the lack of effectiveness is the persistence of autoimmune plasma cells.	Better efficacy of both drugs in comparison with rituximab. Obinutuzumab met endpoints. The study of ocrelizumab was interrupted due to safety concerns.		The primary endpoints were not met, but there was an improvement in symptoms.	The study’s primary endpoints were not met.
Safety	Mild to moderate AEs. The most common AEs are associated with upper respiratory tract infections. There may be a risk of developing allergic reactions due to the chimeric structure of the antibody.	Severe adverse events associated with the development of opportunistic infections.	Mild to moderate AEs.	Mild to moderate AEs.	
Future prospects	It may be advisable to conduct a study in groups of patients with mild to moderate SLE.	There is a low likelihood of subsequent study of the drug due to the increased risk of developing severe AEs.	There is a high probability of conducting phase III clinical trials and approval of the drug.	There is potential to study the effectiveness of these antibodies in other etiological groups of patients with SLE.	

### Antibodies targeting T-cell surface antigens

Due to the fact that the CD40–CD40L signaling pathway is a known pathological pathway in SLE and the cause of the development of lupus nephritis, it is advisable to select one of these biological molecules as a therapeutic target for the development of antibodies. Initially developed antibodies to CD40L caused increased thrombus formation, which is why clinical studies using them were stopped early [27]. Thus, despite the demonstrated effectiveness of the antibody ruplizumab in a phase II study, which was manifested in a decrease in proteinuria, autoantibody titer, and the Systemic Lupus Erythematosus Disease Activity Indexy (SLEDAI)—the shown results persisted even after the study, the study was stopped due to the development of thrombotic effects in several patients, leading to myocardial infarction [45]. In the study [46], the anti-CD40L antibody IDEC-131 (toralizumab) did not show sufficient effectiveness, and a case of thromboembolism was recorded in a patient with Crohn’s disease, which was identified after the study [47].

The cause of thromboembolism when taking monoclonal antibodies to CD40L is considered to be the formation of complexes formed from CD40L antibodies associated with autoantibodies, which can activate platelets through the Fc gamma receptor, leading to aggregation [48]. To prevent this situation, anti-CD40 antibodies have been developed with or without inactive Fc regions and without reduced ability to bind to the target [27]. Thus, when assessing the safety of the antibody dapirolizumab pegol to CD40L without the Fc region, which is replaced by polyethylene glycol (PEG), mostly mild and moderate AEs were recorded, and no cases of thromboembolism were identified [49]. A phase II trial in patients with moderate to severe SLE disease showed significant clinical improvements, but the primary endpoint of the study based on the BICLA assessment was not met, implying that additional studies are needed [50].

ICOS is another promising T-cell target for monoclonal antibody development. The AMG 557 antibody was studied in a clinical trial [51], in which patients with SLE who received AMG 557 had their corticosteroid dose reduced and their immunosuppressive medications completely discontinued. Significant improvements in the treatment of SLE were assessed based on changes in the BILAG and SLEDAI indices. Side effects were mild to moderate. As such, AMG 557 has a high potential for approval for the treatment of SLE following final clinical trials. A comparison of antibodies in development targeting T-cell surface antigens is shown in Table 3.

**Table 3:** Antibodies in development for the treatment of SLE targeting T-cell surface antigens: comparison and analysis

**Antibody**	**Ruplizumab**	**Toralizumab**	**Dapirolizumab pegol**	**AMG 557**
Target	CD40L			ICOS
Efficiency	Reduction of the SLEDAI and improvement of clinical indicators of SLE.	Did not meet the study’s primary endpoints.	Improved clinical indicators of SLE but did not meet the primary endpoint of BICLA.	Improvement of BILAG and SLEDAI indices. Withdrawal of immunosuppressants and reduction of corticosteroids.
Safety	There is a high probability of developing thromboembolism as a result of aggregation of these antibodies with autoantibodies through the Fc gamma receptor.		Mild to moderate AEs, there were no cases of thromboembolism due to Fc site replacement with PEG.	Mild to moderate AEs.
Future prospects	Significant modification of the Fc receptor is required to prevent the risk of thromboembolism.	Low effectiveness and the risk of developing severe AEs do not motivate further research.	It is possible to modify the study design and evaluate effectiveness based on other indices.	High potential for conducting phase II and III clinical trials.

### Antibodies targeting cytokines

There are currently little data from clinical trials of monoclonal antibodies targeting cytokines in SLE. Regarding IL17A, there is a clinical trial that assessed the effectiveness and safety of the AIN457 antibody in the treatment of patients with rheumatoid arthritis (RA), uveitis, and psoriasis [52]. AIN457 was not associated with serious side effects and resulted in improvements in clinical symptoms in all patient groups, confirming the pathological role of IL17 in many inflammatory chronic diseases and encouraging the potential use of AIN457 to treat other related diseases, including SLE.

Ustekinumab is a monoclonal antibody directed against the p40 subunit, which is part of the cytokines IL23 and IL12. A clinical trial [53] showed significant efficacy of ustekinumab in treating patients with moderate to severe Crohn’s disease without severe AEs, which potentially shows the potential for ustekinumab to be used to treat other autoimmune diseases, including SLE. Another monoclonal antibody, briakinumab, which targets the same antigene as ustekinumab, has not shown the required efficacy: it did not reach the primary endpoints in a phase 2b clinical trial of Crohn’s disease, despite the demonstrated remission from its administration and satisfactory safety [54]. In general, ustekinumab showed significantly greater efficacy at lower doses—4.5 mg/kg intravenously already at week 8, in contrast to briakinumab, which was administered at doses of 200, 400, or 700 mg and, despite some efficacy, did not reach the primary endpoint at weeks 12 and 24 of the study. Potential dose increasing to achieve the efficacy of briakinumab does not seem reasonable in this case.

The use of tocilizumab, which targets the IL-6 receptor, showed promising results in a phase I clinical trial in patients with SLE [55]: patients showed a decrease in the Safety of Estrogens in Lupus Erythematosus, National Assessment (SLENA) index by more than 4 points, as well as a decrease in the level of autoantibodies to dsDNA and a decrease in the concentration of autoimmune plasma cells. Tocilizumab was associated with a high incidence of infections, most of which were mild in severity. In view of this, tocilizumab appears to be a promising drug for the treatment of SLE. A comparison of cytokine-targeting antibodies under development is shown in Table 4.

**Table 4:** Cytokine-targeting antibodies in development for SLE: comparison and analysis

**Antibody**	**AIN457**	**Ustekinumab**	**Tocilizumab**
Target	IL17	IL23 and IL12	IL6 receptor
Efficiency	Indicated in patients with RA	Indicated in patients with Crohn’s disease	Reduced SLENA, improved clinical indicators
Safety	Indicated in patients with RA	Indicated in patients with Crohn’s disease	Mild to moderate AEs (increased incidence of mild infections).
Future prospects	Due to the proven significant role of IL17 and IL23 in the pathogenesis of SLE and the success of these antibodies in clinical trials studying the treatment of other autoimmune diseases, there is potential for the use of these antibodies for the treatment of SLE with preliminary clinical trials.		High probability of conducting phase II and III clinical trials.

## Discussion

As can be seen from the above analysis, there are many potential monoclonal antibodies for the treatment of SLE at various stages of development. Unfortunately, we cannot cover all available developments on this topic and have chosen the most interesting in our opinion. This review shows both examples of successful and unsuccessful developments. The emphasis here was not on showing the most effective and safe monoclonal antibodies for the treatment of SLE but on explaining how, based on clinical studies that showed unsatisfactory results, analogous antibodies can be developed but with modified properties that improve their clinical effectiveness and will eliminate the severe AEs that have arisen.

The most promising among the antibodies being developed is obinutuzumab, targeting CD20, which could be approved for clinical use within the next 3–5 years if phase III clinical trials are successfully completed. Future prospects (possible entry into clinical use within 10 years) are seen in the antibodies AMG 557 to ICOS, dapirolizumab pegol to CD40L and tolylizumab, aimed at the IL 6 receptor. Expansion of the arsenal of approved drugs based on monoclonal antibodies for the treatment of SLE may promote better efficacy of therapy (for example, when using a combination therapy of several antibodies acting on different targets) and provide an analog in case of ineffectiveness or severe side effects of the therapy used. The issue regarding the safety of the use of monoclonal antibodies, namely, their immunosuppressive effect, requires further study since all the antibodies shown in this review were characterized by AEs associated with the development of infectious diseases of varying severity.

A promising development option in the current field is the study of hybrid (fusion) proteins therapeutic properties. For example, the telitacicept protein was developed, which is a fusion protein consisting of transmembrane activator and calcium modulator and cyclophilin ligand interactor (TACI) associated with human IgG [56]. Telitacicept is able to block two targets associated with B-lymphocyte maturation: BAFF and APRIL [56], which is its functional advantage compared to belimumab, which targets exclusively BAFF. In a phase I clinical trial, telitacicept in the dose range of 80–240 mg demonstrated safety and tolerability: there were no severe side effects (no AEs associated with respiratory infections were reported), antidrug antibodies were detected in only 2 out of 36 subjects [57]. Also, no serious AEs were shown in a phase 2b clinical trial [58]. This study also demonstrated the efficacy of telitacicept. The proportion of patients achieving SRI-4 response was 68%–75% compared to 34% in the placebo group [58], which is the best indicator for SRI-4 response in belimumab efficacy studies. In addition, significant responses were achieved in all secondary endpoints, including a reduction in the SLEDAI index by >4 points [58]. Thus, telitacicept may potentially represent a better alternative in the treatment of SLE compared to belimumab, especially for patients at high risk of developing infectious diseases. The development of such fusion proteins is a promising task in this field.

Targeting two antigens at once may have an important advantage in the treatment of a multifactorial disease such as SLE. There is currently no this type of drugs approved for clinical use for the treatment of SLE and other autoimmune diseases. However, there are cases showing the potential effectiveness of using bispecific antibodies to treat autoimmune diseases [59], [60]. In addition, this year, the FDA has got application for the phase 1 clinical trials conduction to evaluate the safety, pharmacokinetics, and immunogenicity of the bispecific antibody CLN-978 aimed at the treatment of moderate to severe SLE [61]. CLN-978 targets CD19 on the surface of B cells and CD3 on the surface of T cells and is designed, like other antibodies with a similar bispecific structure, to engage T lymphocytes in targeting autoimmune B lymphocytes [62]. An advantage of CLN-978 is its ability to bind to CD19 on B lymphocytes in extremely low quantities (picomolar concentrations) [62], which potentially indicates a low EC50 of CLN-978 and the ability to achieve the desired effect at low doses. This, in turn, implies a lower likelihood of developing side effects from taking CLN-978.

## Conclusions

Anifrolumab is the most effective approved antibody for the treatment of SLE, but it carries a risk of opportunistic infections that the other approved drug, belizumab, does not have, but is less effective, which may be partly due to the choice of antibody target. Modification of the antibody can reduce side effects and increase efficacy—for example, the development of dapirolizumab pegol. Obinutuzumab is the most promising drug from the group of monoclonal antibodies in the treatment of SLE to enter clinical practice. Antibodies with longer-term prospects include AMG557, dapirolizumab pegol, and tolilizumab. Additionally, the hybrid protein telitacicept and the bispecific monoclonal antibody CLN-978 have high therapeutic prospects.

## Institutional review board statement

Not applicable.

## Data Availability

Not applicable.
